# Care management of patients with high cardiovascular risk in Hungary an international and Hungarian longitudinal comparison of target level achievement

**DOI:** 10.1186/s12875-020-01150-9

**Published:** 2020-05-08

**Authors:** Zoltán Jancsó, Imre Rurik, László Kolozsvári, Lajos Mester, Anna Nánási, Csaba Oláh, Tímea Ungvári, Katalin Vraukó TCs, László Kalabay, Péter Torzsa

**Affiliations:** 1 Department of Family and Occupational Medicine, Faculty of Public Health, University of Debrecen, Debrecen, Hungary; 2 Institute of Family Medicine, Faculty of Medicine, University of Szeged, Szeged, Hungary; 3 Central and University Teaching Hospital of Borsod-Abaúj-Zemplén County, Miskolc, Hungary; 4 Department of Family Medicine, Faculty of Medicine, Semmelweis University, Budapest, Hungary

**Keywords:** Cardiovascular risk, Cardiovascular prevention, Diabetes type 2, Dyslipidemia, EUROASPIRE, High risk patients, Hungary, Hypertension, Primary care, Target level

## Abstract

**Background:**

Patients with high cardiovascular risk are usually cared for in primary care settings. Assessment of the effectiveness of long-time care was a subject of many European studies in the last two decades. This paper aims to present two Hungarian primary care cross sectional surveys and to compare their results to the primary care arms of the European Action on Secondary and Primary Prevention by Intervention to Reduce Events (EUROASPIRE) III. and IV. studies.

**Methods:**

Between 2010 and 2011, 679 patients with high cardiovascular risk were recruited in 20 Hungarian primary care practices and 628 patients were selected in 40 practices between 2015 and 2016. The actual national recommendations were used for classification, all based on European guidelines. Achievements of target levels for blood pressure, total-, LDL-and HDL-cholesterols, triglyceride, and HbA_1c_ (in diabetics) were recorded and analyzed. Further cardiovascular risk factors, such as smoking, BMI, waist-circumference were also evaluated.

**Results:**

There was a statistically significant improvement in the management of blood-pressure and plasma LDL-cholesterol levels among high risk patients, while there was no change in the plasma triglyceride values. The effectiveness of diabetes care deteriorated. In international relation, the management of blood pressure and plasma LDL-cholesterol values were better in Hungary when compared to the results of EUROASPIRE III-IV. studies, while the previous advantage in diabetes care disappeared. A higher proportion of diabetic patients was above the target values in Hungary than the means of the European surveys. There was a higher proportion of smokers in the Hungarian samples, while the proportion of obese and overweight patients was similar to the European sample.

**Conclusions:**

Primary care has a unique role in cardiovascular prevention. Although many of the patients are managed appropriately, there is a need to improve primary care services in Hungary, giving more competences to GPs in prescription and introducing structural changes in the healthcare system.

## Background

Cardiovascular disease of atherosclerotic origin is among the major causes of death and hospital admissions in middle-aged and older people in Europe. Thus, primary and secondary prevention of these morbidities is a top priority of national public health policies in European countries. During the last two decades various national and international societies developed guidelines for physicians in order to support their preventive and curative practice. The first European guideline of the *Joint European Task Force of the European Society of Cardiology and Other Societies on Cardiovascular Disease Prevention* (JETF) was published in 1994 [1] but the real breakthrough came in 2003 with the third guideline [2], introducing the Systematic Coronary Risk Evaluation (SCORE) method. The SCORE is based on European epidemiological data, focused on global cardiovascular risk, estimating the chance of fatal cardiovascular events in the upcoming ten years. This guideline defined target values for the management of major risk factors and gave recommendations on lifestyle change and preventive medication. Currently the sixth recommendation of the JETF [3] is applicable, published in 2016.

Assessment of the success of the implementation of the guidelines and the effectiveness of the continuous care of patients with high cardiovascular risk was the subject of several European studies in the past two decades. The *European Action on Secondary and Primary Prevention by Intervention* to *Reduce Events* (EUROASPIRE-I) survey started in the middle of the 90s, followed by others, involving 12 and 14 European countries. The last EUROASPIRE studies were conducted in 2006–2007 (III.) and in 2014–2015 (IV) respectively, and both of them had primary care arms [4, 5]. In these cross-sectional surveys the study populations were high risk patients without an established cardiovascular disease. Lifestyle (eating habits, physical activity and smoking), BMI, blood pressure and metabolic parameters (plasma lipids, glucose) were recorded and compared to the target values of the relevant JETF guidelines [2, 6]*.* There were limited national publications about what happened in primary care settings and Hungary has not been invited to participate in these studies, although some national surveys were conducted on primary and secondary prevention during this period. Regarding the target values of lipid parameters, the Hungarian REALITY and Multi-GAP studies concluded that the effectiveness of outpatient clinics of lipid specialists or cardiologists was significantly higher than that in primary care, but these differences have gradually diminished over the years [7–9].

The national” *Hungarian Cardiovascular Consensus Conference*s*”* (HCCC) are organized biannually, where opinion leaders of cardiovascular and metabolic diseases discuss and share their experiences. They are followed by the publication of updated guidelines, which are almost identical to the European guidelines of Task Forces [6, 10–12].

We conducted two nationwide cross-sectional surveys to assess the efficacy of cardiovascular risk management in respect of the achievement of target values of some anthropometric, clinical and laboratory parameters in different subgroups of high risk patients in Hungarian primary care. The first survey was performed in 2010–2011, the next one in 2015–2016. In this report our aim was to make two comparisons. The first, between our two surveys referred to above – to follow the changes in efficacy in Hungarian risk management -, and the second, between the results of our surveys and the data of the subpopulation of the relevant European surveys – to compare our results to, i.e. the outcomes of primary prevention arms of EUROASPIRE III. and IV. studies, performed almost at the same time as our surveys [4, 5].

The Hungarian primary health care system is mainly based on solo practices, where one GP and one practice nurse work, and in some cases there is one more assistant. It’s an old and fragmented structure with limited possibilities and resources for patient education and lifestyle management, without real cooperation between the practices and other stakeholders in providing primary and secondary prevention (e.g. health improvement offices, local governments etc.) This situation results in a huge gap between possibilities and needs regarding preventive activities in primary care.

Nowadays there are governmental initiatives in Hungary to create general practice partnerships involving more healthcare professionals and higher resources to support the general risk assessment of patients and risk management activities.

## Methods

Our study group - based on the Hungarian primary care research network - performed a representative nationwide *cross-sectional survey* in 2010–2011. It was repeated in 2015–2016 using almost the same method while taking into account the updates of the national and international guidelines.

Followed by an advertisement for targeted – mostly tutorial – practices, the applicant practices were selected by location and the population they served.

Twenty primary care practices participated in the first survey and 40 in the second one. There were minimal overlaps between the practices, which covered the whole country geographically. Half of the practices were in urban settings, the other half in rural areas.

Patients between 40 and 80 years of age were included to whom one or more of the following medications had been prescribed at least for 12 months before enrollment into the survey: (1) antihypertensive and/or (2) lipid lowering and/or (3) antidiabetic medication. The first 10 persons who visited the primary care office were consecutively selected from each diagnostic (prescription) group. Exclusion criteria were: (1) the available electronic health records were incomplete, (2) participation in our former survey.

All measurements were retrieved from the electronic medical records for each patient, which were: (1) mean values of the last three blood pressure (BP) measurements, (2) laboratory data of fasting values of plasma glucose, HbA_1c_, triglycerides, total, HDL and LDL-cholesterols, based on calculation and (3) anthropometric parameters [13]*.*

Risk assessments were done centrally by the research group according to the IV. and VI. Guidelines of the *Hungarian Cardiovascular Consensus Conferences* (Table 1) [11, 12]. The principal assessment criteria of these guidelines were essentially identical to those of the IV. and V. JETF guidelines [6, 10].

**Table 1:** Cardiovascular risk classifications according to the relevant Hungarian guidelines [11, 12]

2009	2015
**Very high risk:**	**Very high risk:**
• **Coronary heart disease +** o Diabetes mellitus (type 2 or type 1 with micro- or macroalbuminuria) or o Metabolic syndrome or o Smoking	• **Atherosclerotic coronary, cerebrovascular or peripheral artery disease** • **Acute coronary syndrome, ischemic stroke, critical limb ischemia** • SCORE ≥ 10%/10 years • Diabetes mellitus (type 1 or 2) and ≥ 1 further major risk factor and/or target organ damage • Severe chronic kidney disease (eGFR < 30 ml/min/1.73 m^2^ and proteinuria) • Familiar hypercholesterolaemia
**High risk (with CVD or equivalent):**	
• **Atherosclerotic coronary, cerebrovascular or peripheral artery disease** • Diabetes mellitus (type 2 or type 1 with micro- or macroalbuminuria) • Chronic kidney disease	
**High risk without cardiovascular disease:**	**High risk:**
**Subclinical atherosclerosis:** **Atherosclerotic plaque (proven by ultrasound/CT/MRI) without clinical symptoms** Ankle-brachial index ≤0.9 Presence of at least one of these serious risk factors: • Total cholesterol > 8.0 mmol/l • BP > 180/110 mmHg • BMI > 40 kg/m^2^ • eGFR < 60 ml/min • Micro- or macroalbuminuria, proteinuria • Premature cardiovascular event in close relatives (men: < 55 ys, women: < 65 ys) • Left ventricular hypertrophy • SCORE: ≥5% /10 ys • Metabolic syndrome	• **Atherosclerotic plaque (proven by ultrasound/ CT/MRI) without clinical symptoms** • Ankle-brachial index ≤0.9 • Diabetes mellitus (type 1 or 2) without further major risk factors • Chronic kidney disease (eGFR: 30–60 ml/min/1.73 m^2^ and/or proteinuria) • Premature cardiovascular event in close relatives (men: < 55 ys, women: < 65 ys) • BP > 180/110 mmHg • Atherogenous dyslipidemia • BMI > 40 kg/m^2^ • Metabolic syndrome • SCORE: ≥5 < 10%/10 ys
**Medium risk:** SCORE: 2–4%/10 ys	
**Low risk:** SCORE: 0–1%/10 ys	

Abbreviations: *BMI* body mass index, *eGFR* estimated glomerular filtration rate, *CT* computer tomography, *CVD* cardiovascular disease, *MR* magnetic resonance imaging, *RR* blood pressure, *SCORE* systematic coronary risk evaluation

*Statistical analysis* was performed on the relevant data of the two Hungarian surveys by using Student’s unpaired t-test. The level of significance was set to *P* < 0.05. All analyses were performed using the STATA 10.1. software (Statacorp LP. College Station, TX, USA).

Data were stored under the provisions of the Hungarian national health data protection law. According to recent Hungarian regulations, no permission of an ethical committee was required because all data were drawn from patients’ files and were anonymously sent to the data processing center.

All health care providers contributed on a voluntary basis, without any financial incentives.

## Results

Table 2 presents the data of the two Hungarian surveys (2010–2011 and 2015–2016). Sample size and mean values were very similar. Patients with high risk but free from cardiovascular disease (subgroups A and D), reached the target blood pressure in 75.8 and 60%, respectively, which is a significant difference. In the first survey only 37.1 and 30.1% of the patients were below the target threshold (subgroups B and C). This rate was 42.7% in the identical subgroup (E) during the second survey, while the target value had changed from 130/80 mmHg to 140/90 mmHg. Only 33.3% of the patients reached the LDL-cholesterol target value (< 2.5 mmol/l) in the first survey, while five years later it increased to 39.3%. Just over 10% of patients reached the LDL-cholesterol target value (< 1.8 mmol/l) in the first and 15.8% in the second survey, the difference is significant. Reaching the 1.7 mmol/l plasma triglyceride target value was more successful in subgroups B and C. Achievement of the HDL-cholesterol target value improved slightly by the second survey, it was between 64 and 71% in all subgroups.

**Table 2:** Distribution of the Hungarian study populations by categories, by achievement of target values, based on effective national guidelines [11, 12]

Study period	2010–2011			2015–2016	
National guideline launched	**2009**			**2015**	
Total study population (N)	**679**			**628**	
By genders (N)	Male: 320	Female:359		Male: 312	Female:316
≤60 years	141	133		119	92
> 60 years	179	226		193	224
Average age [years±SD]	62.3 ± 11.5			62.7 ± 10.8	
Years under treatment, means [±SD]	8.7 ± 6.4			10.2 ± 7.4	
BMI > 25 kg/m^2^ [%]	81.8			83.9	
BMI > 30 kg/m^2^[%]	40.6			44.4	
waist circumference (men: > 102 cm, women: > 88 cm) [%]	64.3			62.0	
Smoking [%]	21.6			21.0	
	**STUDY SUBGROUPS**			**STUDY SUBGROUPS**	
	**A**	**B**	**C**	**D**	**E**
Subgroups by risk	**ASY**	**HR-CVD**	**VHR**	**HR**	**VHR**
*BLOOD PRESSURE*					
Target value [mmHg]	< 140/90	< 130/80	< 130/80	< 140/90 DM: < 140/85 Nephropathy+proteinuria: < 130/80	
Proportion of patients on target [%]	75.8^*^	37.1	30.1	60.0^*^	42.7
*LDL-CHOLESTEROL*					
Target value [mmol/l]	< 3.0	< 2.5	< 1.8	< 2.5	< 1.8
Proportion of patients on target [%]	47.5	33.3	10.7^*^	39.3	15.8^*^
*TRIGLICERIDE*					
Target value [mmol/l]	< 1.7			< 1.7	
Proportion of patients on target [%]	38.5	55.2	42.1	47.8	45.0
*HDL-CHOLESTEROL*					
Target value [mmol/l]	> 1.0 (male) > 1.3 (female)			> 1.0 (male) > 1.3 (female)	
Proportion of patients on target [%]	71.6	65.6	64.1	66.7	68.0
*DIABETES*					
Target value [HbA_1c_ [%]	< 7.0			< 7.0	
Proportion of patients on target [%]	42.0			40.0	

Abbreviations: *ASY* Asymptomatic high risk patient; *DM* diabetes mellitus; *HR* High risk patients, *HR-CVD* High risk patients with CVD; *VHR* Very high risk patients;

^*^: Significant (*p* < 0.05) difference between the marked subgroups in the same row

In both surveys the glycated hemoglobin (HbA_1c_) target values were analyzed only among type 2 diabetic patients. During the five-year period, there were no improvements in Hungary. The proportion of patients with HbA_1c_ < 7% was 42% in the first and 40% in our second survey, respectively.

Table 3 compares the main findings of the Hungarian surveys to the European studies performed in the same time-period. The measurement of total cholesterol was later replaced by plasma LDL-cholesterol, therefore these values were compared. It was found that the treatment of hypertensive patients was always more effective in Hungary. Total cholesterol levels were almost identical, while LDL-cholesterol level was treated more effectively in Hungarian primary care. Considering only the HbA_1c_ values, the advantage of the Hungarian patients disappeared, five years later the EUROASPIRE data were better [4, 5]. Statistical differences between the Hungarian and European results could not be calculated because of the lack of detailed European datasets.

**Table 3:** Comparison of the laboratory results of subgroups of high-risk patients without CVD in the Hungarian surveys in primary care and European surveys with arms in general practice, study fields, effective guidelines

	I^st^ survey (marked with A in Table 2.)	EUROASPIRE III.	II^nd^ survey (marked with D in Table 2.)	EUROASPIRE IV.
Study period	**2010–2011**	**2006–2007**	**2015–2016**	**2014–2015**
Study field	Hungary	12 European countries	Hungary	14 European countries
Base of risk assessment	Fourth Joint European Task Force Guideline		Fifth Joint European Task Force Guideline	
Risk level	High-risk without CVD		High-risk without CVD	
*BLOOD PRESSURE*				
Target value [mmHg]	< 140/90	< 140/90	< 140/90 (with DM: 140/85)	< 140/90 (with DM: 140/80)
Patients on target [%]	75.8	36.1	60.0	44.7
*TOTAL-CHOLESTEROL*				
Target value [mmol/l]	< 5.0	< 5.0		
Patients on target [%]	33.9	33.7		
*LDL-CHOLESTEROL*				
Target value [mmol/l]			< 2.5	< 2.5
Patients on target [%]			39.3	32.7
Risk level	High risk DM patients without CVD			
*HbA*_*1c*_				
Target value [%]	< 7	< 7	< 7	< 7
Patients on target [%]	48.8	39.9	43.0	58.5

Abbreviations: *CVD* cardiovascular disease, *DM* diabetes mellitus

## Discussion

### Main findings

There was a significant improvement in Hungary in the management of blood-pressure and LDL-cholesterol level among high risk patients. The effectiveness of diabetes care deteriorated.

In international relation, management of blood pressure and LDL-cholesterol values were better in Hungary, while previous advantage in diabetes care disappeared. Higher ratio of diabetic patients was above the target laboratory values in Hungary than the means of the EUROPEAN survey, with no respective national data available from the participating countries.

Other comparisons were made according to the main risk factors and registered laboratory parameters regarding target levels.

### Study population

The Hungarian study population was geographically representative, more patients were involved in the surveys than in other European countries (109 the lowest, 519 the highest), the mean age was 1–2 years higher [4, 5].

### Smoking

The rate of current Hungarian smokers did not change significantly in the high-risk populations (21.6% vs. 21.0%). This ratio is lower than the value of the total adult Hungarian population (29%), but higher than the European average of general population (16.6%) [5, 14].

### Obesity (BMI and waist circumference)

There was an apparent increase in the proportion of overweight (BMI above 25 kg/m^2^: 81.8% vs. 84.3%) and obese people (BMI over 30 kg/m^2^: 40.6% vs. 44.4%). However, this failed to reach statistical significance.

The European survey provided similar findings [5]. The situation is just slightly better regarding waist circumference. The increase in body weight towards pathological levels is described as a real threat to the population of Hungary [15, 16]*.* Unhealthy nutritional habits and the sedentary lifestyle are considered as the main factors responsible [17, 18]. An international comparison has revealed that the situation is not better in Europe, either. The primary care data of EUROASPIRE IV. found that 83.4% of the study populations of the 14 European countries had a BMI over 25 kg/m^2^ [5]*.* Important international recommendations focus on the prevention of obesity in primary care recommending changes in lifestyle and nutrition [3, 19]. This could also be realistic in Hungary, as proven by a previous pilot project within the initiatives of managed care [20].

At the time of the first survey, there were yet three subgroups of high-risk patients (asymptomatic, high risk with cardiovascular disease and very high risk) but only two (high risk and very high risk) at the second evaluation due to the changes of risk classification in the guideline of the VI. *Hungarian Cardiovascular Consensus Conference* [12]. In accordance with the EUROASPIRE III. and IV. primary prevention arms [4, 5], these results were compared to the values of our surveys’ high risk subgroups free from cardiovascular diseases.

### Blood pressure

In all surveys, the Hungarian achievements were better than the European ones; 75.8% vs. 36.1% [4], and 60% vs. 44.7% [5]. The ratio of appropriately treated hypertensive patients decreased significantly (from 75.8 to 60.0%) within the subgroups *free from cardiovascular diseases* (A and D, in Table 2), but improved (to 42.7%) in subgroups with *cardiovascular disease* or with *very high risk* (B, C and E, in Table 2). The latter improvement could be explained with the higher recommended threshold (130/80 vs. 140/90 mmHg) [12]. In our previous study focusing only on hypertension, the target value achievement was more favorable than the European results [9].

### Plasma lipid parameters

In the earlier studies - when only *total-cholesterol* levels were compared - Hungarian data (33.9%) were almost the same as the European ones (33.7%) [4]. Regarding *LDL-cholesterol,* the target value achievements improved significantly in all relevant subgroups. The success rate changed from 33.3 to 39.3% in the *high-risk* subgroup (target: ≤ 2.5 mmol/l), and from 10.7 to 15.8% in the *very high-risk* subgroup (target: ≤ 1.8 mmol/l), respectively, the latter being statistically significant (*p* < 0.05). The target level achievement in the *high-risk subgroup* was better than in the latest European survey (39.3% vs. 32.7%) [5]. These results indicate that “*lipid-management*” is not appropriate in either Europe or in Hungary.

Within the subgroups, the success rates by *triglyceride* were between 38 and 55%, and in case of *HDL-cholesterol* they were between 64 and 72% without significant changes over the five–year period. Primary care examinations of EUROASPIRE III. and IV. did not report detailed European data of these lipid parameters.

### Diabetes care (type 2)

In European comparison - in subgroups without CVD -, data of our first survey were better than the European average (48.8% vs. 39.9%), but this advantage disappeared within five years and the trend turned worse (43% vs. 58.5%) [4, 5].

There is a wide variety of available therapeutic options in primary care settings. All newest and evidence-based medications can be prescribed in Hungary, although GPs have less competence in prescribing some innovative and expensive drugs, mainly in diabetes care. The compliance and adherence of patients are often bad, partially explaining the therapeutic failures*.* Better results in the achievement of blood pressure target values and worse efficacy in performing the target values in lipid-lowering and antidiabetic therapy can be partly explained by the need of deep lifestyle changes in the treatment of type-2 diabetes and the bad statin adherence of patients with dyslipidemia [21, 22]. To improve lifestyle modification and drug adherence we need more human resources and capacities in primary care.

Solo practices dominate in Hungary, i.e. usually one doctor works with one nurse. One of the possible ways for professional improvement could be the cooperation between practices, supported by other health care workers, e.g., dietitian, physiotherapists, health-psychologist or more practice nurses. In this field, a short-term pilot project has been introduced in Hungary [23]. According to the pilot’s results there are new governmental initiatives to support general practice partnerships to be formed where preventive services (health promotion, risk assessment, lifestyle modification, patient education etc.) can be provided in a wider range with higher resources, involving much more healthcare professionals and service providers in a cooperative way. It can result in an improvement in patients’ health behavior, lifestyle changes and adherence to therapies, overall in the reduction of cardiovascular risk. Our conclusions are in line with those of the EUROASPIRE IV. primary care arm study in European context [5].

### Study limitation

The comparison of different, perhaps not representative populations in different countries, at different times, with different competences at primary care level could be the most important limitations of our study. However, the goals and methods used in the management of patients with high cardiovascular risk are based on common European guidelines.

The criteria systems referring to subgroup classification and the number of subgroups have been changed in the previous decade [11]. In some categories, the target values were also modified. These changes made the analyses more difficult, but we tried to compare the results of the most similar subgroups in both Hungarian and international respects.

The international overview could be the strength of this study and the achievements of the Hungarian GPs are ready for international comparison.

## Conclusions

Primary care has a unique role in cardiovascular prevention in each European country. The effectiveness of cardiovascular risk management in Hungary did not improve essentially during the period between 2010 and 2016. Family physicians should be helped by updated education on more effective methods in patients’ education and lifestyle intervention with an approach of cooperative management in general practice partnership settings. They need more support with the involvement of other health care professionals, wider community orientation and expanded competencies. Medical advice against smoking, inappropriate nutritional habits, sedentary lifestyle and obesity should be promoted both in the general population and at governmental levels.

## Data Availability

The datasets used and/or analyzed during the current study are available from the corresponding author on reasonable request.

## Abbreviations

ASY: Asymptomatic
BMI: Body mass index
CT: Computer tomography
eGFR: Estimated glomerular filtration rate
EUROASPIRE: European Action on Secondary and Primary Prevention by Intervention to Reduce Events
GPs: General practitioners
DM: Diabetes mellitus
HbA_1c_: Glycated hemoglobin
HDL-cholesterol: High density lipoprotein cholesterol
HCCC: Hungarian Cardiovascular Consensus Conference
HR: High risk
HR-CVD: High risk with cardiovascular disease
JETF: Joint European Task Force of the European Society of Cardiology and Other Societies on Cardiovascular Disease Prevention
LDL-cholesterol: Low density lipoprotein cholesterol
MRI: Magnetic resonance imaging
SCORE: Systematic Coronary Risk Evaluation
VHR: Very high risk
